# The aging immune system and all-cause mortality in older americans: differences across sex and race/ethnicity

**DOI:** 10.1186/s12979-025-00521-z

**Published:** 2025-06-21

**Authors:** Kate A. Duchowny, Yuan S. Zhang, Rebecca C. Stebbins, Xin Ma, Jaydon Jun Yu Chin, Virginia W. Chang, Allison E. Aiello, Grace A. Noppert

**Affiliations:** 1 Institute for Social Research, University of Michigan, 426 Thompson St, Ann Arbor, MI 48104 USA; 2 Department of Sociomedical Sciences, Mailman School of Public Health, Columbia University, 722 W 168th St, New York, NY 10032 USA; 3 Robert N. Butler Columbia Aging Center, Mailman School of Public Health, Columbia University, 722 W 168th St, New York, NY 10032 USA; 4 Department of Biostatistics, Mailman School of Public Health, Columbia University, 722 W 168th St, New York, NY 10032 USA; 5 Inland Revenue Authority of Singapore, Singapore, Singapore; 6 Institute for Social and Economic Research and Policy & Mailman School of Public Health, Columbia University, 722 W 168th St, New York, NY 10032 USA; 7 Department of Social and Behavioral Sciences, School of Global Public Health, New York University, 708 Broadway, New York, NY 10003 USA; 8 Department of Population Health, Grossman School of Medicine, New York University, 708 Broadway, New York, NY 10003 USA; 9 Department of Epidemiology, Mailman School of Public Health, Columbia University, 722 W 168th St, New York, NY 10032 USA

**Keywords:** Population health, Immunosenescence, Social determinants, Disparities, Mortality

## Abstract

**Background:**

As individuals age, the immune system undergoes complex changes, including an increase in the number of CD8 T cells relative to CD4 T cells, a decline in naïve cell production (including T and B cells), and an accumulation of terminally differentiated cells with diminished functionality. These age-related immune alterations collectively contribute to immunosenescence, a phenotype associated with aging-related declines and diseases such as dementia, Alzheimer’s disease, osteoporosis, and diabetes. Premature mortality at older ages often results from cumulative health deterioration initiated by physiological dysregulation over the life course. Mortality risk, therefore, provides a meaningful measure of the long-term impact of physiological changes, including those related to the immune system. Examining the link between mortality risk and immune aging in older adults could illuminate the underlying pathology of aging-related health decline. This study uses data from the Health and Retirement Study (HRS), a national, population-based sample of middle-aged and older Americans, to explore the relationship between specific immune aging ratios and six-year mortality, stratified by race/ethnicity and sex.

**Results:**

Using a sample of 8,259 individuals from the HRS, we found that overall, the presence, magnitude, and direction of the association differed by the specific immune ratio measure, sex, and race/ethnicity. We found particularly robust associations among Hispanic and non-Hispanic Black females. Among Hispanic females, for example, a one-unit increase in the log CD4 EMRA: Naïve ratio was associated with a nearly 50% increase in mortality for Hispanic females and a 25% increase in mortality for non-Hispanic Black females which was robust to adjustment for additional covariates. While we found little evidence of an association between immune function and mortality among non-Hispanic White and Hispanic males, we found associations in the opposite direction as what we would expect among non-Hispanic Black males. For example, a one-unit increase in the CD4, EMRA: Naïve ratio was associated with a 15% decrease in mortality among non-Hispanic Black males.

**Conclusions:**

Our findings demonstrate that associations between immune aging and mortality are not uniform but instead vary in magnitude and direction across sex and racial/ethnic subgroups. The strongest and most consistent associations were observed among Hispanic and non-Hispanic Black females—groups experiencing multiple forms of marginalization—suggesting that these populations may face heightened vulnerability to the downstream consequences of immune aging. However, the absence or reversal of expected associations in some subgroups—particularly non-Hispanic Black males—underscores the complexity of immune aging processes and their interaction with social and biological contexts. These results highlight the importance of disaggregated analyses and suggest that immune aging may manifest and impact mortality risk differently across populations.

**Supplementary Information:**

The online version contains supplementary material available at 10.1186/s12979-025-00521-z.

## Introduction

The aging of the immune system (or immunosenescence) refers to dynamic age-related changes that occur in immune systems that can lead to a decline in immune function, which has been implicated in a host of negative health conditions [1–3]. Typically, as individuals age, the immune system undergoes complex functional and compositional changes across its various components which are crucial for understanding aging-related decline and disease [4–6]. Notably, aging is associated with an increased ratio of CD8 T cells to CD4 T cells, a decline in the production and number of naïve cells (including T and B cells), and an accumulation of terminally differentiated cells with reduced functionality [4, 7, 8]. These alterations contribute to an aged immune profile characterized, for example, by a diminished pool of naïve T cells and an increase in late-stage, antigen-specific CD8 T cells with limited functional capacity [8, 9]. Chronic herpesvirus infections, particularly cytomegalovirus (CMV), are thought to play a significant role in driving the expansion of antigen-specific T cells [6, 10]. Collectively, these age-related immune changes contribute to a phenotype of immunosenescence, which is linked to a range of other aging-related declines and diseases, including dementia, Alzheimer’s disease, osteoporosis and diabetes [2, 3, 11, 12]. 

More recently, there is increased interest among social scientists in understanding immune aging because, in addition to being thought of an underlying driver of many aging-related conditions, it is also hypothesized to be shaped by physical and social environments across the life course. As such, immune aging may serve as an important pathway by which life experiences and exposures “get under the skin” to influence health and the aging process [13, 14]. Recent studies of population-level trends in immune aging have reported differences in markers of immune aging by sex, race/ethnicity, and education. The differences observed in these studies are equal to, or in some instances, exceed the differences in immune aging typically observed with the normal aging process, indicating that certain subgroups may have profiles of advanced immune aging related to, at least in part, to the social adversity they may have to had to endure [14]. Studies have also consistently shown that disadvantaged individuals have markers of more advanced immune aging compared to their less disadvantaged counterparts. For example, immune profiles indicative of advanced immune aging have been observed among individuals who have experienced post-traumatic stress disorder, have low income or low education, those classified as minority race/ethnicity, and those reporting housing insecurity and incarceration [15, 16]. Similarly, among older adults, living in disadvantaged neighborhoods was found to be associated with markers of advanced immune aging [17, 18]. Taken together, these studies provide initial evidence that immune aging may serve as a biological pathway through which socioenvironmental factors produce disparities in health and aging.

Despite the recognition there are complex and dynamic changes that occur within the immune system as it ages, population health and social scientists have tended to rely on singular measures such CRP or IL-6, or the number of CD4 naïve T cells, which are inadequate metrics to fully understand specific aspects of the immune system and its role in disease etiology. As a result, there has been a recent push to integrate immune ratio measures within ongoing population health studies that include diverse samples because these measures may be more effective in capturing changes to the composition of the T cell compartment to better understand immune aging and its role across several key subpopulations and health outcomes, including mortality.

Premature mortality at older ages is often a consequence of cumulative health deterioration that is initiated by physiological dysregulation that occurs over the life course [19]. As such, mortality risk serves as a salient measure of the long-term impact of multiple physiological changes, including immune aging. Understanding the relationship between mortality risk and immune aging in older adults could provide critical insight into the underlying pathology of aging-related health deterioration. The relationship between mortality and various immune parameters has been reported in previous studies, although findings have been inconsistent. For example, research into a sample of older Swedes aged 86 to 92 years found that those with a combination of high CD8 and low CD4 T cell percentages and poor T cell proliferation in peripheral blood lymphocytes had higher mortality rates [7, 20]. A study of persons of 65 and older with HIV infection reported that a low CD4:CD8 ratio over time was associated with a heightened risk of mortality [21]. However, data from the Newcastle 85 + study found that the ratio of total CD4 to CD8 T cells was not related to all-cause mortality, but frequencies of senescence-like CD4 memory cells were found to be predictive of all-cause mortality. This study also found that senescence in CD4 and CD8 T cell compartments was associated with cardiovascular mortality [2, 5, 22]. Another study that used data from a sample of healthy Italians aged 65 and older reported a positive association between mortality and the absolute number of memory CD8 T cells in female subjects but not in male subjects [23]. Nonetheless, most of the aforementioned studies relied on homogeneous samples from geographically limited areas or selective populations, which restricts the external validity of these findings, underscoring the need for research that considers diverse and representative populations.

In this study, we address the gaps highlighted above by utilizing data from the Health and Retirement Study (HRS), a national, population-based sample of middle aged and older Americans, to examine the relationship between specific immune aging ratio measures and subsequent six-year mortality, stratified by race/ethnicity and sex. Most relevant to the present study are two recent investigations that also used HRS data to examine associations between mortality and T cell ratio measures and T cell subsets. One study found no significant association between two-year mortality and the CD4:CD8 ratio, CD8 Naïve, CD4 Naïve, memory CD4 T cells, or the CD8 Naïve: Memory ratio, but reported a significant association with the CD4 Naïve: Memory ratio [24]. The other study found a negative association between 4-year mortality and total T cells, CD4 Naïve, and memory B cells, but observed a positive association with CD4 terminally differentiated T cells [25]. Our study extends this prior work by examining mortality over an approximately six-year follow-up period and hypothesizes that associations with short-term and longer-term mortality may differ due to biological processes that operate at different time scales, selection in samples, and underlying causes of death. We examine variations by sex and race/ethnicity, considering well-documented evidence of sex-based differences in immune functions, biological aging, and longevity as well as the distinct immune aging profiles of racial and ethnic groups due to long standing social inequities across the life course [13, 26–30]. 

## Data and methods

### Study population

We utilize data from the U.S. Health and Retirement Study (HRS), a national multistage area probability sample of households in the United States, with oversamples of Blacks, Hispanics, and residents from the state of Florida. Details about the HRS sampling strategy can be found elsewhere [31]. The HRS includes more than 22,000 adults over the age of 50 at baseline, and core interviews with HRS participants are conducted every two years [32]. All HRS panel respondents who completed an HRS core interview in the 2016 wave, except proxy respondents and those residing in nursing homes, were asked to consent to a venous blood draw.

Venipuncture was performed during a separate home visit by a trained phlebotomist approximately two months after the core interview [33]. We obtained immune function data for this study from the 2016 Venous Blood Study (VBS). Of the 15,509 eligible HRS participants, 11,974 individuals consented to the VBS, and 9,933 had complete and valid test results, with a consent rate of 78.5% and completion rate of 65% [34]. Our sample includes those individuals who had (1) a valid survey weight, (2) full covariate data, and (3) valid data for immune biomarkers which are required to calculate the immune aging measures used in this study. The final sample size for our study is 8,259, comprised of 2,381 White males, 561 Black males, 502 Hispanic males, 3,161 White females, 927 Black females, and 690 Hispanic females.

### Mortality

The HRS follows respondents at every wave and, at each interview wave, seeks to conduct interviews with earlier respondents. In the HRS, mortality is ascertained from reports of informants in exit interviews. For example, date of death (month/year) is provided by survivors in the HRS exit interview. Weir assessed the completeness of mortality ascertainment by comparing HRS mortality rates to U.S. life tables and concluded that mortality ascertainment in the HRS is effectively complete [35]. In this study, we assessed all-cause mortality between the time of the VBS collection and the 2022 core interviews. For individuals in the analytic sample, the VBS collection occurred between March 2016 and October 2017, and the 2022 core interviews were conducted between March 2022 and September 2023.

### Immunophenotyping methodology

Blood sample collection was conducted in participants’ homes as part of the VBS. Blood was drawn into cell preparation tubes (CPTs) and shipped overnight to the University of Minnesota for processing and testing [34]. Upon arrival, CPTs were centrifuged, and peripheral blood mononuclear cells (PBMCs) were isolated and cryopreserved. Thawing was conducted in batches of 10 samples. Immunophenotyping occurred over the course of 18 months using two flow cytometers [36]. The HRS team implemented multiple quality control measures (described below) to monitor instrument performance, antibody consistency, and inter-technician variability [36, 37]. 

Protocols used to define immune cell subsets followed the Human Immunology Project Consortium guidelines, including the use of CD95 to identify effector cytotoxic T cell subsets [34, 36, 38]. One vial of cryopreserved PBMCs, containing approximately 4 million cells, was thawed and incubated at 37 °C in RPMI media for one hour. Cells were then centrifuged at 1200 rpm for 10 min at room temperature, resuspended in 1× phosphate-buffered saline (PBS), and stained as described in Crimmins et al., 2017 [34]. Cells were subsequently kept on ice until analysis.

Flow cytometry was performed using either an LSRII or Fortessa X20 cytometer (BD Biosciences, San Diego, CA). A hybrid approach was used for immunophenotyping: the OpenCyto R package was employed for automated hierarchical gating, and manual gating using FlowJo (v10, Tree Star Inc.) was conducted on a subset of 931 h participants to validate automated results [39]. Five major immune cell populations were identified: T cells, B cells, monocytes, natural killer (NK) cells, and dendritic cells. Gating was performed on singlets using forward scatter area (FSC-A) versus height (FSC-H), followed by selection of viable lymphocytes based on FSC/SSC characteristics. T and B cells were expressed as a percentage of total lymphocytes, while monocytes, NK cells, and dendritic cells were expressed as a percentage of total PBMCs. Subsets were reported as a percentage of their respective parent population. Absolute cell counts were calculated by multiplying the percentage of each subset by the total lymphocyte count obtained from the complete blood count (CBC) and expressed as 10⁹/L.

To further characterize cytotoxic and helper T cell subsets, three surface markers—CCR7, CD45RA, and CD28—were used to distinguish naïve, memory, and terminally differentiated populations. In total, flow cytometry was used to identify 33 distinct T cell, B cell, monocyte, and NK cell subsets. A full list of which markers were used to define each cell subset is provided by the HRS team and can be found at: https://hrsdata.isr.umich.edu/sites/default/files/documentation/data-descriptions/1636738697/HRS_FlowCytometryDocReport_202111.pdf.

**Quality control (QC) measures.** In addition to standard quality control (QC) procedures, the HRS laboratory team also developed additional QC measures including collecting control samples from healthy volunteers that were cryopreserved at the start of the study and were analyzed at least twice per week using the study protocol to monitor laboratory shifts and drifts in the immunophenotyping assessments [36, 37]. 

### Ratio measures of immune aging

Following previously established methodologies [13, 14, 30], we used the following ratios of T cell phenotypes to represent immunosenescence: CD4: CD8, EMRA: Naive CD4, EMRA: Naive CD8, B cell Memory: Naive.

The EMRA: Naïve CD4, EMRA: Naïve CD8 and the B cell Memory: Naïve ratios are constructed such that higher values for all measures indicate a more aged immune system [14]. The CD4: CD8 ratio is constructed according to the traditional way this measure has been used, and lower values are consistent with a more aged immune system [9, 40]. We log-transformed all ratio measures to approximate normality for our statistical analyses.

### Statistical analyses

We conducted all primary analyses stratified by sex and race/ethnicity. Stratification was chosen a priori to account for known biological differences in immune function by sex, and for systematic differences in health by race/ethnicity that reflect the impacts of structural racism on health over the life course [14, 26, 30]. Stratified models allow all covariates—including the exposure and potential confounders—to vary in their associations with the outcome across groups. To formally assess potential effect measure modification, we additionally tested interaction terms between the immune outcomes*sex and immune outcomes*race/ethnicity in non-stratified models. These results are provided in Table S1. However, we note that interaction models typically assume that only the coefficient for the exposure of interest varies by subgroup, while all other covariates are held constant across groups, which may not be appropriate in this context and thus we proceed with stratified models.

We first present descriptive statistics, followed by a series of three Cox proportional hazards models for each race and sex group in order to assess the hazard of mortality associated with each ratio measure of immunosenescence. We used attained age as the time scale in the Cox models [41]. This approach provides a non-parametric control for age, allowing the baseline hazard to flexibly account for age-related risk without requiring explicit modeling of age as a covariate. This strategy provides a natural framework for assessing time-to-event outcomes in aging populations [42]. We then selected model covariates that could confound the relationship between mortality and immune aging, avoiding those that could be affected by immune function (i.e., mediators) and thus lie on the causal pathway (e.g., diseases, functional limitation, and disability). Model 1 is unadjusted, with attained age as the time-to-event variable. Model 2 adds years of schooling, and Model 3 further includes smoking status. All analyses were weighted using the 2016 VBS weights to account for the complex survey design, nonresponses to the household interview, and nonparticipation in the blood collection [43]. We conducted statistical analyses in R.

### Sensitivity analyses

We conducted several sensitivity analyses, First, we replicated the regression models controlling for CMV IgG. Since prior research indicates that CMV may be a major driver of immune aging [8, 44, 45] we were interested in examining whether the inclusion of CMV would attenuate the association of the immune aging measures on the hazard of mortality. CMV IgG levels were measured in serum using the Roche e411 immunoassay analyzer [34]. Second, we constructed models using a binary indicator of the CD4: CD8 ratio measure comparing a ratio less than one compared to values greater than or equal to 1, consistent with how this variable has been used in previous analyses [9, 40, 46]. Finally, we constructed an additional model (Model 4) that included control variables for inflammatory markers (CRP and IL-6) and number of physician-diagnosed comorbidities (a continuous measure, 0–7), of eight conditions of high blood pressure, diabetes, cancer, lung disease, heart disease, stroke, psychiatric conditions, and arthritis).

## Results

Table 1 presents the sample characteristics stratified by race/ethnicity and sex. Although the mean age at VBS collection was 68.9 for non-Hispanic White males and 70.1 for non-Hispanic White females and slightly lower for non-Hispanic Black and Hispanic males and females, non-Hispanic Black and Hispanic males and females overall showed higher levels of immune aging than their White counterparts across all four immune aging measures. Among males, for example, non-Hispanic White males had the lowest mean value of the log-transformed CD4 EMRA: Naïve ratio (− 3.60, SD = 1.72), compared to − 2.70 (SD = 1.75) in non-Hispanic Black males and − 2.68 (SD = 1.64) in Hispanic males. These values suggest that White males had a greater proportion of naïve CD4 + T cells relative to terminally differentiated cells, indicating a less aged immune profile. Among females, the mean log-transformed CD4 EMRA: Naïve ratio was − 2.57 (SD = 1.66) in non-Hispanic Black females, compared to -2.88 (SD = 1.46) in Hispanic females and − 3.54 (SD = 1.68) in non-Hispanic White females.

**Table 1:** Demographic characteristics of the study sample, stratified by race/ethnicity and sex (*N* = 8,259)

	White male	Black male	Hispanic male	White female	Black female	Hispanic female
	*n* = 2,418	*n* = 561	*n* = 502	*n* = 3,161	*n* = 927	*n* = 690
Ratio measures of immune aging, natural logarithm (ln) transformation, Mean (SD)						
CD4:CD8	1.09	0.98	0.93	1.23	1.05	1.03
	(0.71)	(0.76)	(0.64)	(0.70)	(0.62)	(0.63)
CD4 EMRA: Naïve	-3.60	-2.70	-2.68	-3.54	-2.57	-2.88
	(1.72)	(1.75)	(1.64)	(1.68)	(1.66)	(1.46)
CD8 EMRA: Naïve	0.97	0.72	1.36	0.67	0.53	1.06
	(1.45)	(1.38)	(1.15)	(1.44)	(1.34)	(1.22)
B Cell: Memory: Naïve	-1.79	-1.68	-1.85	-1.90	-1.68	-1.91
	(1.05)	(0.94)	(0.89)	(0.99)	(0.91)	(0.90)
Sociodemographic characteristics						
Age at blood collection	68.92	67.27	67.09	70.08	68.81	68.09
	(8.71)	(8.11)	(8.17)	(9.67)	(9.23)	(8.63)
Educational attainment, %						
Less than high school	6.37	22.74	45.04	7.21	25.31	51.45
High School/GED	29.83	34.74	21.59	33.67	31.81	24.53
Some College	24.49	26.38	20.88	29.95	25.48	16.38
College and above	39.31	16.14	12.49	29.17	17.40	7.63
Smoking status, %						
Never smoker	38.11	31.46	36.82	50.63	44.15	56.01
Former smoker	51.35	42.34	49.40	39.53	38.38	35.84
Current smoker	10.54	26.2	13.78	9.84	17.47	8.15
Mortality						
# deaths	513	109	74	588	135	74
% deaths, weighted	15.24	16.82	14.01	15.59	17.51	11.68
Median follow-up time (years), Alive	5.76	5.65	5.66	5.80	5.70	5.69
Median follow-up time (years), Deceased	3.39	3.26	3.83	3.48	2.94	3.76

Substantial differences in educational attainment were observed across race/ethnicity groups, with non-Hispanic White males and females having a much lower proportion with less than a high school education (6.37% for males and 7.21% for females) and a much higher proportion with a college degree or above (39.31% for males and 29.17% for females) compared to their non-Hispanic Black and Hispanic counterparts, among whom 16.14% of non-Hispanic Black older males, 12.49% of Hispanic older males, 17.4% of non-Hispanic Black females, and 7.63% of Hispanic females had a college degree or higher. Smoking was most prevalent among non-Hispanic Black males and females (26.2% and 17.47%, respectively) and much less prevalent among White males (10.54%), White females (9.84%), and Hispanic females (8.15%). From the time of the VBS collection to the 2022 core interviews, 1,493 deaths were reported. The percentage of deaths was highest among non-Hispanic Black males (16.82%) and females (17.51%) and lowest among Hispanic males (14.01%) and females (11.68%). The median follow-up time was approximately 5.7 years for the living participants and about 3–4 years for the deceased.

## Regression results

Table 2 reports the hazard ratios (HR), *p*-values, and 95% confidence intervals from the weighted Cox proportional hazards models across all race/ethnicity and sex groups. We explored two different methods for modeling associations with the CD4:CD8 ratio. In our primary analyses, we modeled CD4:CD8 continuously, with lower values consistent with a more aged immune system (the opposite direction of the other ratio measures analyzed). Then, in sensitivity analyses we modeled the CD4:CD8 ratio as a binary variable with a threshold at 1.0, as is commonly done with this measure, and we compared those with a value < 1.0 to those with a ratio value greater than or equal to 1.0 (see Table S3).

**Table 2:** Hazard ratios from weighted Cox proportional models predicting mortality. **Model 1** includes age as the time-to-event variable. **Model 2** further adds years of schooling and **Model 3** further includes smoking status. Panel A corresponds to results for males. Panel B corresponds to results for females

Panel A. Results for males									
	Non-Hispanic White			Non-Hispanic Black			Hispanic		
	HR	*p*-value	95% CI	HR	*p*-value	95% CI	HR	*p*-value	95% CI
CD4: CD8									
M1	0.93	0.27	(0.82,1.06)	1.69	0.003	(1.19, 2.38)	1.18	0.54	(0.69, 2.03)
M2	0.92	0.22	(0.82, 1.07)	1.7	0.003	(1.2, 2.40)	1.19	0.52	(0.70, 2.04)
M3	0.93	0.31	(0.82, 1.07)	1.7	0.003	(1.20, 2.40)	1.08	0.81	(0.60, 1.95)
CD4 TEMRA: Naïve									
M1	1.01	0.88	(0.94, 1.08)	0.85	0.064	(0.72, 1.01)	1.05	0.54	(0.90, 1.22)
M2	1	0.93	(0.93, 1.07)	0.86	0.055	(0.73, 1.00)	1.04	0.59	(0.89, 1.22)
M3	1	1	(0.93, 1.07)	0.85	0.033	(0.74, 0.99)	1.04	0.56	(0.90, 1.21)
CD8 TEMRA: Naïve									
M1	0.97	0.29	(0.91, 1.03)	0.74	0.001	(0.62, 0.88)	0.92	0.49	(0.71, 1.18)
M2	0.96	0.28	(0.90, 1.03)	0.73	0	(0.62, 0.87)	0.91	0.43	(0.71, 1.15)
M3	0.97	0.44	(0.91, 1.04)	0.73	0.001	(0.61, 0.88)	0.96	0.8	(0.71, 1.30)
B cell memory: naïve									
M1	1.09	0.15	(0.97, 1.22)	0.84	0.023	(0.72, 0.98)	1.21	0.12	(0.95, 1.53)
M2	1.12	0.05	(1.00, 1.25)	0.82	0.014	(0.70, 0.96)	1.22	0.11	(0.96, 1.56)
M3	1.1	0.095	(0.98, 1.24)	0.81	0.006	(0.70, 0.94)	1.28	0.1	(0.96, 1.72)

**Panel B.** Results for females									
	Non-Hispanic White			Non-Hispanic Black			Hispanic		
	HR	*p*-value	95% CI	HR	*p*-value	95% CI	HR	*p*-value	95% CI
CD4: CD8									
M1	1.01	0.925	(0.88, 1.15)	0.83	0.113	(0.65, 1.05)	0.59	0.002	(0.41, 0.83)
M2	1.01	0.93	(0.88, 1.15)	0.81	0.075	(0.64, 0.11)	0.6	0.002	(0.44, 0.83)
M3	0.98	0.772	(0.86, 1.12)	0.93	0.51	(0.73, 1.18)	0.61	0.005	(0.43, 0.86)
CD4 TEMRA: Naïve									
M1	1.02	0.61	(0.95, 1.08)	1.24	0.026	(1.03, 1.50)	1.45	0.005	(1.12, 1.89)
M2	1.01	0.74	(0.95, 1.08)	1.25	0.031	(1.02, 1.54)	1.45	0.012	(1.09, 1.93)
M3	1.02	0.55	(0.95, 1.10)	1.19	0.059	(0.99, 1.42)	1.47	0.006	(1.12, 1.93)
CD8 TEMRA: Naïve									
M1	0.89	0.004	(0.83, 0.96)	1.07	0.28	(0.94, 1.22)	1.22	0.013	(1.04, 1.44)
M2	0.89	0.004	(0.82, 0.96)	1.08	0.19	(0.96, 1.22)	1.2	0.022	(1.03, 1.41)
M3	0.9	0.01	(0.83, 0.98)	1.04	0.57	(0.91, 1.19)	1.2	0.03	(1.02, 1.41)
B cell memory: naïve									
M1	1.01	0.88	(0.91, 1.16)	1.22	0.063	(0.99, 1.50)	1.35	0.086	(0.96, 1.91)
M2	1	0.99	(0.90, 1.11)	1.23	0.072	(0.98, 1.54)	1.34	0.09	(0.96, 1.88)
M3	1	0.95	(0.91, 1.10)	1.05	0.73	(0.80, 1.39)	1.34	0.088	(0.96, 1.87)

Among males, results for the CD4:CD8 ratio analysis varied by racial/ethnic group and model specification. Among Hispanic males, neither the continuous nor binary operationalization was significantly associated with mortality (see Table 2, Table S3). For non-Hispanic White males, estimates from both specifications were directionally inconsistent and not statistically significant. Notably, among non-Hispanic Black males, the continuous models showed a strong and statistically significant association, with higher CD4:CD8 ratios (i.e. a less-aged immune system) linked to increased mortality risk (HRs: 1.69 (95: CI, 1.19, 2.38), *p* = 0.003, Model 1). However, this finding was not corroborated in the binary models, which showed non-statistically significant associations in the opposite direction (HR: 0.32 (95% CI: 0.08, 1.29, *p* = 0.11)). These discrepancies suggest potential non-linearity in the association with CD4:CD8 or imprecision due to subgroup sample size, and underscore the importance of evaluating immune markers both continuously and categorically when examining mortality risk across diverse populations.

With regards to females, when modeled continuously, the CD4:CD8 ratio was significantly associated with mortality only among Hispanic females. In this group, a lower CD4:CD8 ratio was consistently associated with higher mortality risk across all models (HR: 0.59 (95% CI: 0.41, 0.83); *p*-value: 0.002, Model 1). These findings align with the binary specification of CD4:CD8 (< 1.0 vs. ≥1.0), which also showed statistically significant elevated hazard ratios (HR: 2.46 (95% CI: 1.16, 5.21), *p* = 0.02). A similar pattern was observed among non-Hispanic Black females, though it did not reach statistical significance. There were no associations observed among non-Hispanic White females.

A similar pattern among subgroups emerged for the CD4⁺ EMRA: Naïve ratio. There were no statistically significant associations among males except for non-Hispanic Black males. In Model 3, a 1-unit increase in the log transformed CD4 + EMRA: Naïve ratio was associated with a 15% reduction in mortality risk (HR = 0.85, 95% CI= (0.74, 0.99)) controlling for years of schooling and smoking status. Among females, there were statistically significant associations among both non-Hispanic Black and Hispanic females. For example, among Hispanic females, a 1-unit increase in the log CD4⁺ EMRA: Naïve ratio was associated with a nearly 50% greater mortality risk (Model 1 HR: 1.45, 95% CI = 1.12, 1.89). The adjustment of educational attainment and smoking did not meaningfully change these associations.

For the CD8⁺ EMRA: Naïve ratio, there were no statistically significant associations among non-Hispanic White or Hispanic males. Among non-Hispanic Black males, a 1-unit increase in the log CD8 + EMRA: Naive ratio was associated with a 26% reduced mortality risk as shown among non-Hispanic Black males (Model 1 HR = 0.74, 95% CI = 0.62, 0.88). This result was robust to additional controls for educational attainment and smoking status (Models 2–3). Similarly, among non-Hispanic White females, 1-unit increase in the log CD8 + EMRA: Naïve ratio was associated with an approximate 10% decreased mortality risk (Model 3 HR = 0.90, 95% 0.83, 0.98). In contrast, the CD8⁺ EMRA: Naïve was positively associated with mortality risk for Hispanic females. A 1-unit increase in the log CD8 + EMRA: Naïve ratio was associated with 20% higher mortality risk (Model 3 HR = 1.20, 95% CI = 1.02, 1.41).

Regarding the B cell Memory: Naïve ratio, there was evidence of a modest association with mortality among non-Hispanic White males in Model 2 (HR = 1.12, 95% CI = 1.00, 1.25) and no statistically significant association for Hispanic males. Conversely, a significant but inverse association was observed among non-Hispanic Black males (Model 3 HR = 0.70, 95% CI = 0.70, 0.94). Among females, there are no statistically significant associations observed between the B cell Memory: Naïve ratio and mortality risk.

### Sensitivity analyses

In sensitivity analyses, we estimated whether the addition of CMV would attenuate the observed associations. Overall, we found that the results remained largely unchanged. See Table S2. We also constructed an additional model (M4) that included other control variables for inflammatory markers and comorbid conditions. Of note, these variables may also be on the causal pathway from immune function to mortality and thus M4 may represent an over-controlled model. Generally, we found that neither the direction of the effects nor the statistical significance of the association did not change in M4. Results are presented in Table S4.

## Discussion

Using a national population-based cohort, we found statistically significant associations between four markers of immune system aging and six-year all-cause mortality: CD4:CD8 Ratio, CD4 EMRA: Naive Ratio, CD8 EMRA: Naive Ratio, and the Ratio of Memory: Naïve B Cells. Overall, the presence, magnitude, and direction of the association differed by the specific immune ratio measure, sex, and race/ethnicity. Generally, among males, we observed weaker and fewer statistically significant associations between the immune aging ratio measures and mortality. With regards to non-Hispanic Black males, however, we observed associations in the opposite direction than we expected in which profiles consistent with a more aged immune system were associated with decreased mortality risk. Among females, the associations varied substantially by racial/ethnic subgroup. Among females who self-identified as non-Hispanic Black and Hispanic, we observed a strong, positive association between immune aging and mortality whereby a more aged immune profile was associated with increased risk of mortality. Taken together, our findings demonstrate that associations between immune aging and mortality are not uniform but instead vary in magnitude and direction across sex and racial/ethnic subgroups. While we do see evidence that how the immune system ages may contribute to physiological deterioration that is associated with premature mortality, the absence or reversal of expected associations in some subgroups underscores the complexity of the immune aging process and the ways in which it may be shaped by social and biological contexts. Our results highlight the importance of disaggregated analyses and suggest that immune aging may impact mortality risk differently across populations.

While the literature on population-level immune aging is still developing, prior studies have reported an association between immune aging and multiple health outcomes such as disability, cancer, and diabetes [30, 47, 48]. In addition, emerging evidence suggests that differential immune system aging may contribute to many of the persistent health inequities (e.g., by socioeconomic status and race/ethnicity) observed in aging-related decline and disease. Although we did not formally test mediation in this study, our findings are consistent with the hypothesis that immune aging could be one biological pathway linking social adversity to premature mortality. Specially, we observed the strongest associations between immune aging and mortality among non-Hispanic Black and Hispanic older females—groups that consistently experience multiple forms of structural marginalization across the life course. While a formal mediation analysis is beyond the scope of the current investigation, these results highlight the potential importance of immune aging as a link between the social and the biological, and support the continued research aimed at disentangling pathways leading to health inequities. To further help contextualize these subgroup differences we posit several biological and social explanations below that may provide context in understanding our study findings.

From a biological standpoint, immune system differences between males and females emerge early in life and persist across the lifespan [49–51]. On average, females have higher CD4 T cell counts than males of the same age [14, 16, 28, 49, 50]. In contrast, males tend to have higher CD8 T cell counts [49]. Additionally, both human and mammalian studies suggest that biologic males may mount more robust immune responses to pathogens earlier in life, which may be particularly prominent in the innate immune compartment and associated with accelerated immune aging and immune exhaustion by late life among males [50]. In contrast, biological females typically exhibit stronger antibody responses across the life course, including in late life, which may contribute to observed differences in immune function and mortality risk [49, 52]. B cell dynamics are also modified by sex; [53, 54] females tend to have higher B cell counts in general and across the life course [51], which may further contribute to sex differences in immune aging. It is well established that, although females typically have stronger immune responses, they also become more susceptible to autoimmune and inflammatory diseases with age [49, 55]. Therefore, collectively this prior work suggests that there may be greater variability in immune system composition and function among females compared to males which help to explain why we observed more robust associations among females [56]. Future research should examine immune aging earlier in the life course in order to examine how these immune parameters may evolve over time for each sex and to what extent they are associated with premature mortality.

The associations we observed between the immune aging measures and mortality among non-Hispanic Black and Hispanic women must be considered in relation to the social and structural factors that have been repeatedly shown to put minoritized groups at increased risk for poor health across the life course. Prior research has consistently found that social and structural determinants of health shape aging trajectories and disease risk over the life course [57, 58]. Women, and particularly women of color, experience greater cumulative exposure to stress and trauma, which may accelerate immune system aging and lead to premature mortality [59–61]. Despite having lower overall mortality rates and greater longevity, women tend to experience a higher burden of chronic disease and disability [62, 63]. This same trend held true in the current investigation: Hispanic females, for example, had the lowest mortality rate of any subgroup, but the association between immune aging and mortality was consistently strongest in this group.

Counterintuitively, we observed associations in the opposite direction for Black males. One potential explanation for this finding may be due to selection effects. In other words, since our analytic sample was composed of individuals who were healthy enough to be enrolled in the HRS study, individuals who died or became ill would not have been able to participate. Prior research indicates that contemporary older adults of different racial/ethnic backgrounds were born and have survived under varying age-specific mortality patterns with Black individuals experiencing significantly higher mortality rates at all ages, particularly at younger ages, compared to their White counterparts [64]. Thus, it is possible that sicker individuals may not have entered into the HRS cohort, thus biasing our effect estimates away from the null and showing “protective” effects when in fact selection processes may serve as an alternative explanation. Again, examining trajectories of immune aging earlier in the life course may lend key information about how divergent aging trajectories emerge among subgroups of the population.

We also observed that the presence, direction, and strength of associations varied across the different immune aging ratio measures, which is consistent with prior research indicating that these measures capture different aspects of immune system aging and reflect distinct biological processes at both the cellular- and systems-level. The ratios examined in this study capture two major shifts in the immune system that are both compositional and functional, as noted in the introduction. Both shifts may contribute to the mortality associations observed in our study. For example, CD8 (cytotoxic) and CD4 (helper) T cells play distinct roles in immune function. CD8 T cells primarily target virus-infected or tumor cells, whereas CD4 T cells coordinate immune responses by activating cytotoxic T cells, B cells, and aspects of the innate immune system [65–67]. CD8 T cells are known to reach a terminally differentiated state more rapidly than CD4 T cells, potentially resulting in less variability among older adults and weaker associations with mortality [67]. In contrast, CD4 T cells remain functionally diverse, playing multiple roles in immune signaling and regulation, which may explain why the composition of the CD4 cells (e.g., as captured by CD4 EMRA: Naive ratios) was more strongly associated with mortality outcomes [66, 68]. 

While our results add to a growing body of evidence demonstrating the importance of understanding immune system aging, this study has several limitations. First, there were aspects of the laboratory procedures that may have introduced measurement error. For example, immunophenotyping was conducted over the course of 18 months, with samples thawed and analyzed in batches of ten [36]. As a result, the duration of cryopreservation varied across participants. Although all samples were cryopreserved under uniform conditions and standard protocols were followed, variability in storage time may influence the expression of certain surface markers. This is particularly relevant for subset-specific ratios such as EMRA: Naïve or Memory: Naïve T and B cell populations. Unfortunately, detailed information on the duration of cryopreservation for individual samples is not available, and we were unable to account for this variability in our analyses. In addition, immune aging was assessed at one point in time, thus precluding our ability to make inferences about the pace of immune aging and how that may contribute to mortality. Related, these single point in time assessments do not capture any dynamic feedback relationships that may occur between the immune measures over the life course. Immune aging unfolds beginning at birth and there are important dynamics throughout this process we are unable to capture by looking at a single point in time. While our application of multiple immune measures represents a significant step forward towards a more holistic assessment, we nonetheless relied on a limited set of immune measures that capture only a small portion of the immune system. Future studies should continue to move towards a systems-based approach for understanding immune aging to better capture complexities in these processes. With regards to the outcome, we examined all-cause mortality in the present investigation. It may be that immune aging is most relevant for specific causes of death. Future work should explore whether associations between immune aging markers and mortality vary by specific causes of death, particularly those with strong immunological underpinnings such as cardiovascular disease or cancer.

## Conclusion

Despite these challenges, our study offers several important strengths, including the utilization of a large, national cohort of older adults from diverse racial and ethnic backgrounds, the use of biologically-specific markers of immune aging, and high-quality mortality follow-up. We found that more advanced immune aging—particularly as measured by the CD4 EMRA: Naïve and CD4:CD8 ratios—was associated with increased mortality risk in several subgroups, most notably among non-Hispanic Black and Hispanic females. However, we also observed unexpected patterns, including inverse associations between immune aging and mortality particularly among non-Hispanic Black males. These findings underscore the complexity of immune aging processes and suggest that their impact on mortality may vary across sociodemographic subgroups. While this study does not formally test immune aging as a mediator, the results are consistent with the hypothesis that immune aging may represent one biological pathway through which social disadvantage shapes health outcomes in later life. Understanding this pathway is critical for advancing health equity and may inform future interventions aimed at mitigating long-standing disparities in aging and mortality.

## Electronic supplementary material

Below is the link to the electronic supplementary material.

Supplementary Material 1

## Data Availability

The dataset(s) supporting the conclusions of this article is(are) available through the publicly available Health and Retirement Study repository, 10.1093/ije/dyu067 and https://hrs.isr.umich.edu.
