# Supplementary material for: The aging immune system and all-cause mortality in older americans: differences across sex and race/ethnicity
Source: Immun Ageing. 2025 Jun 21;22:25. doi: 10.1186/s12979-025-00521-z (PMC12181923; doi:10.1186/s12979-025-00521-z)
Supplement: Supplementary file 1 — Supplementary Material 1 [file 12979_2025_521_MOESM1_ESM.docx]

**Supplemental File**

**Table S1.** Results of the interaction tests estimating the presence of a statistical interaction between the immune outcomes and sex, and race/ethnicity.

|  | **P value** | **P value** | **P value** |
| --- | --- | --- | --- |
| **Outcome** | **Outcome*Female** | **Outcome*Black** | **Outcome*Hispanic** |
| CD4:CD8 | 0.4 | 0.07 | 0.26 |
| CD4, EMRA:Naïve | 0.16 | 0.72 | 0.02 |
| CD8, EMRA: Naïve | 0.96 | 0.09 | 0.31 |
| B, mem:naïve | 0.99 | 0.31 | 0.05 |

**Table S2.** Hazard ratios from weighted cox proportional models predicting mortality. **Model 1** includes age as the time-to-event variable and CMV IgG. **Model 2** further adds years of schooling and **Model 3** further includes smoking status. Panel A corresponds to results for males. Panel B corresponds to results for females.

**Panel A.** Results for males

**Panel B.** Results for females

**Table S3.** Hazard ratios from weighted cox proportional models predicting mortality using the binary classification of the CD4+:CD8+ Ratio. **Model 1** includes age as the time-to-event variable. **Model 2** further adds years of schooling and **Model 3** further includes smoking status. Panel A corresponds to results for males. Panel B corresponds to results for females.

| 1. **Males** |  |  |  |  |  |  |  |  |  |  |  |  |  |
| --- | --- | --- | --- | --- | --- | --- | --- | --- | --- | --- | --- | --- | --- |
|  |  |  | Non-Hispanic White | | |  | Non-Hispanic Black | | |  | Hispanic | | |
|  |  |  | HR | p-value | 95% CI |  | HR | p-value | 95% CI |  | HR | p-value | 95% CI |
|  | CD4: CD8 Binary (less than 1.0 compared to greater than or equal to 1) | | | | | | | | | | | | |
|  |  | M1 | 1.11 | 0.58 | (0.77, 1.61) |  | 0.32 | 0.11 | (0.08, 1.29) |  | 1.38 | 0.45 | (0.53, 2.69) |
|  |  | M2 | 1.19 | 0.31 | (0.85, 1.66) |  | 0.32 | 0.1 | (0.08, 1.17) |  | 1.15 | 0.73 | (0.51, 2.60) |
|  |  | M3 | 1.19 | 0.31 | (0.85, 1.67) |  | 0.31 | 0.08 | (0.08, 1.17) |  | 1.38 | 0.45 | (0.60, 3.19) |
|  |  |  |  |  |  |  |  |  |  |  |  |  |  |

| 1. **Females** |  |  |  |  |  |  |  |  |  |  |  |  |
| --- | --- | --- | --- | --- | --- | --- | --- | --- | --- | --- | --- | --- |
|  |  | Non-Hispanic White | | |  | Non-Hispanic Black | | |  | Hispanic | | |
|  |  | HR | p-value | 95% CI |  | HR | p-value | 95% CI |  | HR | p-value | 95% CI |
| CD4: CD8 Binary (less than 1.0 compared to greater than or equal to 1) | | | | | | | | | | | | |
|  | M1 | 0.92 | 0.69 | (0.61, 1.40) |  | 1.21 | 0.55 | (0.64, 2.29) |  | 2.46 | 0.02 | (1.16, 5.21) |
|  | M2 | 0.95 | 0.8 | (0.63, 1.42) |  | 1.32 | 0.41 | (0.68, 2.55) |  | 2.35 | 0.003 | (1.10, 5.03) |
|  | M3 | 1.02 | 0.9 | (0.69, 1.52) |  | 1.27 | 0.41 | (0.72, 2.25) |  | 2.3 | 0.042 | (1.03, 5.15) |

**Table S4.** Hazard ratios from weighted cox proportional models predicting mortality comparing Model 3 to Model 4. **Model 3** includes age as the time-to-event variable, the immune outcome, education and smoking status. **Model 4** further adds CRP, IL-6, and an index of chronic conditions. Panel A corresponds to results for males. Panel B corresponds to results for females.
